# The Public Health Implications of Online Food Delivery Platforms: A Narrative Review

**DOI:** 10.1007/s13679-026-00771-y

**Published:** 2026-10-30

**Authors:** Adyya Gupta, Rebecca Bennett, Anna Peeters

**Affiliations:** 1 Deakin Centre for Global Preventive Health and Nutrition, Deakin Institute for Health Transformation, School of Health and Social Development, Deakin University, Geelong, Australia; 2 Nutrition and Health Innovation Research Institute, School of Medical and Health Sciences, Edith Cowan University, Joondalup, WA Australia; 3 Victorian Health Promotion Foundation, VIC, Australia

**Keywords:** Digital food environment, Online food delivery, Public health, Diet, Physical activity, Policy, Review

## Abstract

**Purpose of Review:**

This review summarises current knowledge on the role of online food delivery (OFD) platforms in shaping dietary behaviours, sedentary behaviours and other related health outcomes.

**Recent Findings:**

OFD platforms predominantly promote and facilitate access to energy-dense, nutrient-poor foods. Drivers such as convenience, time scarcity, and algorithmic personalisation reinforce unhealthy eating patterns by making less healthy options more visible and accessible. Evidence concerning sedentary behaviour remains sparse. A small number of studies suggest that OFD use may be displacing traditional food procurement behaviours resulting in reduced physical activity. OFD platforms further influence health through environmental, labour, and corporate activities.

**Summary:**

Despite their growing influence, OFD platforms remain largely unregulated within existing policy frameworks. There is an urgent need for interdisciplinary research, improved methodological rigour, regulatory change, and industry accountability to mitigate the health risks associated with OFD platforms and harness their potential for promoting healthier diets and related health behaviours.

## Introduction

Global rates of overweight and obesity have been steadily rising [1], attributed in part to the increasingly “obesogenic” nature of modern food environments [2]. Two key features of modern obesogenic food environments are the widespread availability and consumption of unhealthy foods, such as food high in saturated fats, added sugars, and sodium, and reduced opportunities for physical activity, resulting in increased sedentary behaviours. Unhealthy diets and sedentary behaviours are key modifiable disease risk factors for overweight and obesity, which may lead to increased risk of non-communicable diseases (NCDs) including cardiovascular disease, some cancers, as well as increased risk of premature mortality [3, 4].

Contemporary modern food environments are comprised of both physical and digital (or online) components [5]. While physical food environments and how they impact consumers diet and physical activity have been studied for many years, our understanding of how digital food environments influence health is still emerging [6]. Digital food environments include digital spaces where consumers engage with food online through purchasing, marketing, entertainment, and information sharing across digital platforms [5]. One of the most rapidly expanding segments of the digital food environment is online food delivery (OFD) platforms (also referred to as “meal delivery apps” in literature), where customers can use a smartphone app to order food from restaurants, takeaways, and other retailers for delivery.

A small number of multinational corporations dominate the OFD sector, generating billions of dollars in annual revenue. Globally, in 2024, revenue generated by major OFD platforms ranged from approximately US$34 billion by Meituan (primarily operates in China), US$13 billion by Uber Eats (operates in 45 countries), US$10 billion by DoorDash (operates in the United States, Canada, Australia, Japan, and New Zealand), US$3 billion by Just Eat Takeaway (operates in over 20 countries), US$2 billion by Deliveroo (operates primarily in Europe, the Middle East, and Asia-Pacific) and US$1.5 billion by GrabFood (operates in Southeast Asia) to US$2 billion by Zomato (operates in India and the United Arab Emirates (UAE)) [7]. The sector overall is predicted to generate over US$520 billion in global revenue by 2030 [8]. Market research analysis estimates that markets with high penetration rates of OFD platforms include the United States (USA) (75% penetration rate), China (74%), United Kingdom (UK) (69%), Germany (57%), and Italy and Brazil (both 49%) [9]. Additionally, the global user base in 2025 was estimated to be over 2.2 billion people [10]. A recent cross sectional analysis of survey data from the USA, UK, Australia, Canada and Mexico found that 36% of the sample (*n* = 19,877) reported using OFD platforms in the previous 30 days [11], while another study reported that average number of meals purchased using OFD platforms nearly doubled between 2018 and 2021 [12].

This rapid growth and widespread adoption of OFD platforms have significant implications for public health. These platforms are reshaping how people access, purchase, and consume food, with potential consequences for dietary quality, energy intake, and broader lifestyle behaviours (including sedentary behaviours). At the same time, their expansion raises questions about social and environmental impacts, as well as regulatory oversight. Despite their growing role in everyday food practices, the health implications of OFD platforms remain poorly understood.

To address this gap, this review adopted a narrative approach to synthesise current evidence on the health and societal implications of OFD platforms. Although not a systematic review, relevant literature was identified through targeted searches of academic sources. Studies were critically appraised for methodological rigour, relevance, and contribution to public health evidence before inclusion in the synthesis. The review critically evaluates emerging evidence on the influence of OFD platforms on diet, physical activity, and other health-related outcomes, and aims to illustrate how policy and research are responding to this rapidly expanding digital food environment to mitigate the influence of OFD platforms on health and society.

## Impact of OFD Platforms

### Dietary Impacts of OFD Platforms

Dietary impacts are a central focus of current research on OFD platforms, reflecting growing concern that these platforms may promote energy-dense foods resulting in increased purchase and consumption of these foods. Evidence from multiple countries indicates a consistent pattern of high availability and promotion of unhealthy food options on OFD platforms as discussed below.

A cross-sectional analysis of 13,841 menu items from Uber Eats in Sydney, Australia found that over 80% of items were discretionary [13] (discretionary foods refer to foods and drinks that are nutrient-poor, energy-dense i.e. high in saturated fats, sugars, salt and/or alcohol and not essential for a healthy diet) when coded to the Australian Dietary Guidelines [14]. A similar study in New Zealand using comparable methods found that 73% of menu items (*n* = 25,877) were discretionary, most commonly classified as “cereal based mixed meals” [15]. This study also found that menu items that were discretionary were more likely to also be categorised by the platform as “most popular” (OR: 2.0, 95% CI 1.7–2.2), include a photo of the item (OR: 1.7, 95% CI 1.6–1.8), or offered as part of a value bundle (OR: 4.6, 95% CI 3.2–6.8) [15]. Another study [16] illustrated a real-time account of a diverse range of digital marketing techniques prevalent on OFD platforms operating in Australia. These include: the presence of, and strategic placement of selected food products, price discounts and promotion strategies. The study further demonstrated participants’ engagement with these marketing strategies through multiple clicks to reduce the total cost of their final OFD order. This indicates how subtle nudges towards commonly promoted unhealthy food items at multiple points during the purchase influence the food choices made on OFD platforms.

Another New Zealand study, analysing menu items from Uber Eats and Menulog (a subsidiary of Just Eat Takeaway), reported a “strong bias” in OFD platform menus towards unhealthy items [17]. This study also found that a high proportion of unhealthy food was offered in areas with greater socioeconomic disadvantage [17]. Similar findings were observed in a study from Victoria, Australia, where area-level socioeconomic disadvantage and rurality were associated with decreased outlet healthiness, as measured using a digital food environment scoring tool [18]. A study from the UK using artificial intelligence-enabled analysis of menu items from Just Eat (a subsidiary of Just Eat Takeaway) found an inverse dose-response association between area deprivation and menu healthiness [19]. Supporting this, a large scale analysis of 71,532 food outlets in the UK found that menu prices were generally lower in areas of greater socioeconomic deprivation, with price promotions most common in outlets selling unhealthy or discretionary foods [20]. A recent systematic review found also that unhealthy menu items on OFD platforms were more likely to feature promotional marketing techniques such as price promotions, priority placement at the top of search results, or items bundled as “combo deals” [21].

Evidence from other international contexts further reinforces these concerns. A laboratory nutritional content assessment of popular restaurants across three Thai OFD platforms found that most meals were unhealthy and exceeded daily recommendations for sodium and sugar [22]. In Brazil, a longitudinal study over 16 weeks found that most of the 1,593 food items promoted on OFD platforms through price discounts, photos and taste statements were unhealthy [23]. A cross-sectional analysis comparing OFD platform availability across Chicago (USA), Melbourne (Australia), and Amsterdam (The Netherlands), found that all cities most frequently offered food from fast-food outlets selling pizza and burgers [24]. Two cross-sectional studies conducted in Kochi and Ahmedabad (two cities in India) reported that frequent OFD use was significantly associated with a preference for unhealthy foods, increased portion sizes and perceived changes in body weight among working professionals in India [25, 26]. In China, analysis of OFD menus and survey data revealed an inverse relationship between menu item healthiness and popularity among university students [27].

In addition to quantitative findings, qualitative evidence suggests that consumers are conscious of nutritional issues when using OFD platforms. For example, in the UK, participants expressed how they expected food available through OFD platforms to be unhealthy, and did not use these services with the intention of purchasing healthy food [28]. Similarly, participants in an Australian study reported often selecting less healthy meals but expressed enthusiasm for platform design features that could help identify healthier options [29].

### Physical Activity Impacts of OFD Platforms

Evidence examining the relationship between OFD platform use and physical activity remains sparse and methodologically limited, although early findings suggest potential implications for sedentary behaviour. By replacing activities such as grocery shopping, meal preparation, or walking to food outlets, OFD platforms may contribute to more sedentary patterns of daily living. However, the direction of this relationship remains unclear.

A cross-sectional survey of 409 consumers in China found that a sedentary lifestyle was positively associated with continued use of OFD platforms, with habitual use and the intention to continue using these services strongly predicting actual use behaviour, and sedentary lifestyle further influencing these relationships [30]. Conducted in the post-pandemic era, this study highlights that greater reliance on OFD platforms may persist beyond the restrictions of COVID-19 [30]. Similarly, another cross-sectional survey of employees in China (*n* = 328) found that sedentary behaviour was positively associated with actual use of OFD platforms, and that for those with higher sedentary behaviour, the intention to use these services was less important because they were already more likely to use them [31]. Supporting these findings, a perspective piece on the health impacts of China’s rapid urbanisation and economic growth theorised that shifts in food purchasing habits brought about by OFD platforms could further reduce opportunities for physical activity [32]. However, both studies relied on self-reported data on behaviour, shared similar limitations in design, and were conducted in a single country with a highly developed OFD sector, which limits generalisability. Notably, the only study from outside China reported the opposite. A cross-sectional study of Australian adults (*n* = 2010) found a positive relationship between using an OFD platform at least once in the previous month, and meeting physical activity guidelines (self-reported ≥ 150 min/week of moderate and vigorous physical activity in the past week) [33]. This finding likely reflects confounding by age, as the most common users of OFD services are generally younger people [12] who are also more likely to meet physical activity guidelines [34].

Overall, the evidence linking OFD platform use to physical activity or sedentary behaviour remains extremely limited, inconsistent, and largely speculative. At present, no causal conclusions can be drawn, and further research, particularly longitudinal or device-measured physical activity studies, is needed before meaningful claims can be made.

### Other Related Impacts

Beyond dietary and behavioural outcomes, OFD platforms are embedded within broader food systems that shape environmental sustainability, labour conditions, and corporate power. Although the evidence base is limited, emerging studies highlight several concerns.

#### Environmental Sustainability Impacts

The environmental sustainability impacts of OFD services are increasingly recognised, with emerging evidence pointing to their contributions to packaging waste, carbon emissions, and food system inefficiencies. To address these concerns, in China, platforms like Meituan and Ele.me have launched large-scale sustainability programs. Meituan’s Green Mountain Initiative (2017) [35] released 4.15 million eco-friendly packages and recycled 29,000 tonnes of plastic containers. In 2022, Ele.me launched smaller portions, reduced disposables, and recycling initiatives, and recorded 1.1 billion “no cutlery” orders, saving 58,000 tonnes of CO₂ emissions [36]. A 2021 Australian study [37] estimating greenhouse gas emissions associated with food packaging from OFD services found that emissions from an order ranged from 0.15 to 0.29 kg of CO_2_e, primarily from producing the raw materials necessary to manufacture packaging. The same study also calculated that the annual emissions associated with OFD platform food packaging were expected to grow by 132% by 2024, reflecting the sector’s rapid growth and limited environmental regulation. Similarly, a 2021 case study of OFD drivers in London, UK, found that greenhouse gas emissions varied greatly depending on the mode of transport they used, with cars and moped deliveries having greater emissions than those made by bicycles [38]. The study also noted that because online food deliveries are highly individualised, they are less efficient per tonne delivered than other forms of food distribution [38]. Despite these findings, consumer awareness and concern appear limited. A 2024 case study of experiences with OFD platforms in Poland and Italy (*n* = 600) found that consumers generally disagreed that OFD platforms had a negative environmental impact, and showed limited support for pro-environmental behaviour related to OFD services [39]. This represents a disconnect between the public perception and the environmental footprint of OFD services.

#### Labour Conditions

OFD services also shape contemporary labour markets through their reliance on gig economy models, which offer flexibility for workers but are often associated with income insecurity, limited protections, and precarious working conditions. Sociology scholars have called the employment conditions of OFD drivers a form of “flexible exploitation”, wherein promises of autonomy and flexibility mask underlying economic vulnerability through their classification as independent contractors [40]. This critique is supported by a 2025 case study of Uber Eats in Australia, which found that reliance on inexpensive gig economy workers was a key part of Uber’s business strategy [41]. The physical and psychological demands of this work are also well documented, with a UK study reporting that drivers experienced distraction, fatigue, and pressure to violate traffic rules in order to meet delivery times, with many reporting daily “near misses” in traffic [42]. Similarly, a cross-sectional survey of OFD platform delivery drivers (*n* = 248) in Colombia found that fatigue was significant among riders, increasing their risk of crashes [43].

#### Corporate Power

Finally, the expansion of OFD services exemplifies broader trends in corporate concentration, with a small number of multinational companies exerting growing influence over food systems, data infrastructures, and consumer choices. The negative influence of large companies over the food system has been well documented [44–46], driven by their obligation to maximise shareholder profits, often at the expense of public health. In the OFD sector, market dominance by a small number of powerful companies (Uber Eats, DoorDash and Just Eat), alongside partnerships with multinational companies such as Alibaba, Amazon, Google and Meituan, has created a highly concentrated digital marketplace [47–49]. This concentration may enable conditions where these platforms are able to control how the sector operates [50], potentially reducing competition, increasing consumer prices, and reinforcing asymmetries in access to consumer data and in decision-making power. Such concentration not only shapes market power and pricing but also limits transparency and accountability in how platforms curate menus, promote products, and use consumer data, which are factors with direct implications for dietary health and equity.

**Fig. 1:**
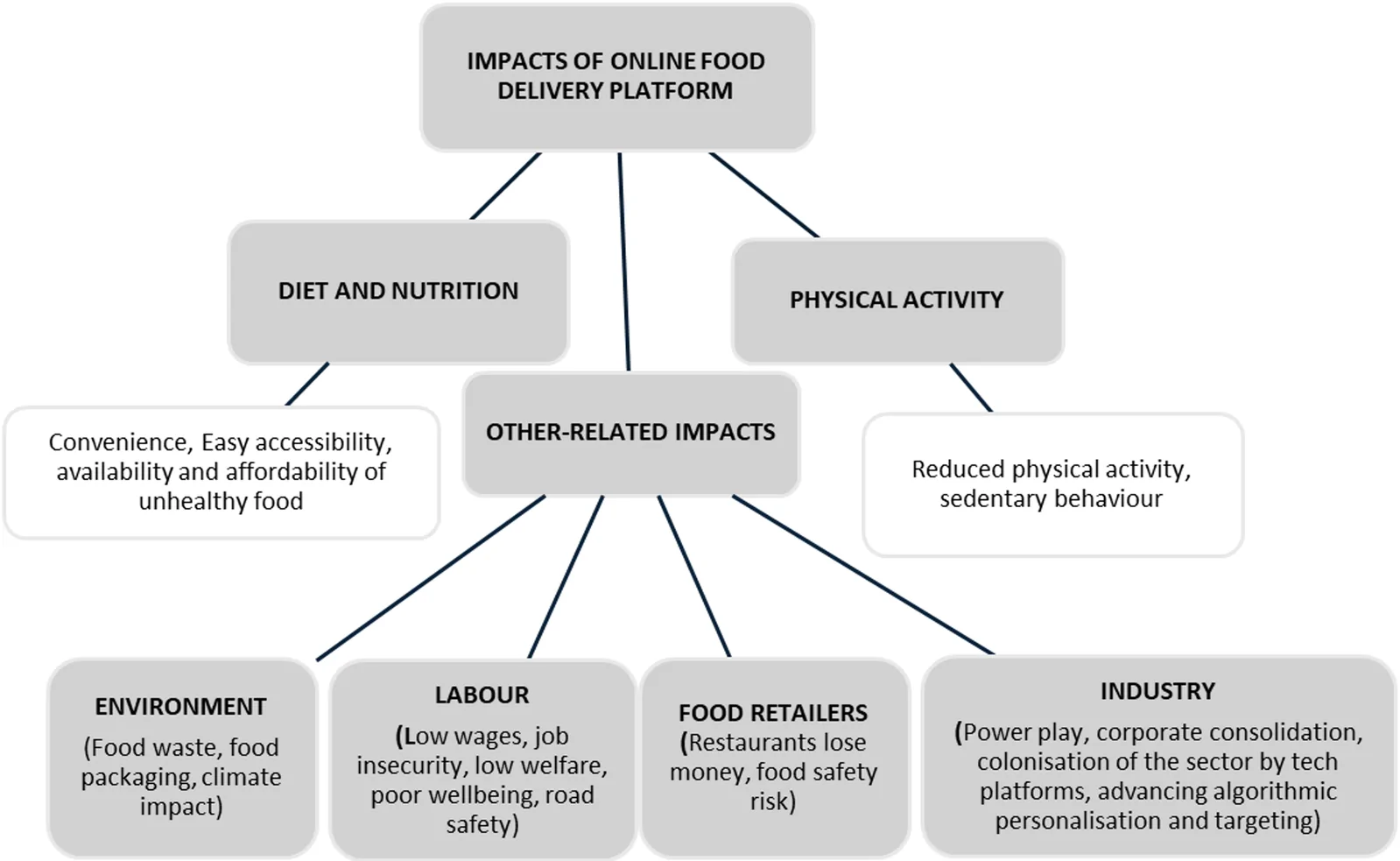
Impacts of online food delivery platforms

Overall, these environmental, labour, and corporate dynamics underscore that OFD platforms have the potential to influence health not only through diet, but also through the broader systems in which food is produced, delivered, and governed (Fig. 1). Understanding these indirect pathways is essential for developing comprehensive policy responses that extend beyond nutritional considerations alone.

## Policy Landscape and Regulatory Gap

### Current Nutrition and Food Environment Policies Aligned to OFD Platforms

As digital food environments become increasingly central to food choice and dietary behaviours, regulating OFD platforms is a public health priority. The World Health Organization has framed “digital food environments” as online settings that shape food choices—an umbrella that clearly encompasses OFD services, and identified them as a priority area for policy action to address diet-related non-communicable disease risk [51]. Yet, a 2024 mapping review concluded that OFD platforms are rarely included in existing nutrition policies, with only a small number of jurisdictions explicitly covering platforms across labelling, promotion and composition [52]. Some countries have taken steps towards integrating digital environments in existing regulations. In the United States, FDA menu-labelling rule (in effect since May 7, 2018) requires chain restaurants (≥ 20 locations) to display calories on menus and menu boards; guidance and local enforcement materials explicitly state this includes electronic menus and internet menus (online order menus) [53]. In England, the Calorie Labelling (Out of Home Sector) Regulations 2021 mandate calorie display by large out-of-home businesses (≥ 250 employees) from 6 April 2022, with implementation guidance covering on-premise and take-away contexts including delivery channels [54]. In Australia, several states and territories (e.g., NSW, QLD, SA, ACT, VIC) have legislated kilojoule menu labelling for large fast-food chains [55], and recent NSW guidance (2025) [56] reiterates the program’s aim to support informed choices across ready-to-eat retail, while federal consultations have acknowledged the relevance of online ordering and web-based platforms. The UK currently has placement restrictions for physical food retail and has pledged to implement restrictions on advertising and promotion including online advertising of unhealthy food and beverages from 2026 onwards [57]. Other countries have adopted voluntary approaches; Singapore’s ‘Healthier Dining Programme’ [58] allows food and beverage companies to offer three or more healthier food or beverage options on online platforms and receive endorsement from the Health Promotion Board. These examples illustrate that while some components of nutrition policy can be adapted to OFD platforms, comprehensive and consistent regulation remains limited.

### Challenges in Regulating OFD Platforms

Although many nutrition related policies can improve population diets and prevent NCDs [59], applying these to OFD platforms presents regulatory challenges. These include: [1] classification ambiguity which creates uncertainty about which actors bear responsibility for nutrition disclosures and marketing practices [2], the use of advanced, personalised and targeted digital marketing techniques that exploit consumer data and operate with limited transparency, and [3] resistance from industry, resulting in weak accountability and limited enforcement.

#### Classification Ambiguity

Unlike traditional food outlets, OFD platforms position themselves as intermediaries rather than food retailers, yet they influence consumer food choices through menus, prices, promotions, and search rankings. This hybrid role places them in a regulatory “grey zone”; it is unclear whether they should be treated as retailers, food service providers, or logistics companies [60]. In practice, many regulations bind the food business (restaurant/chain) rather than the aggregator platform, creating responsibility gaps when menus are distributed across multiple online platforms and applications with inconsistent or missing nutrition information. For example, existing frameworks (e.g., UK Out-of-home labelling [54], US menu-labelling [53]) principally target food outlets, thereby leaving OFD platforms outside explicit nutrition policy unless they are specifically named in policy. As a result, accountability for nutrition disclosures, promotions, and product claims becomes fragmented, complicating enforcement and weakening consumer protections.

#### Digital Marketing Techniques

OFD platforms leverage sophisticated, data-driven marketing strategies that draw on consumer’s personal information such as demographics, geographic location, browsing patterns, and purchase history to personalise menus, search rankings, and promotional offers [16, 61]. These techniques enable highly targeted promotions of high‑margin discretionary and ultra-processed foods, making them easily accessible and highly visible through defaults, deals and recommendations, often undermining public health objectives. Limited transparency around data use and algorithmic decision-making raises ethical concerns regarding manipulation, consent, and the potential for reinforcing unhealthy consumption behaviours, particularly among vulnerable populations such as young people. Furthermore, there is some evidence that competition dynamics between retailers [62] can push offerings toward energy‑dense, nutrient‑poor options as some menus show high proportions of unhealthy items and greater promotional exposure than others.

#### Resistance from Industry

The OFD sector is dominated by a small number of powerful multinational corporations with significant political influence, which can impede regulatory progress [41]. As seen in other areas of nutrition policy, industry resistance often manifests through self-regulation initiatives that lack enforceability, as well as efforts to dilute or delay mandatory standards (e.g., debates on scope/exemptions for high in fat, sugar, or salt advertising on OFD platforms) [63]. This creates accountability and monitoring gaps, where compliance relies heavily on less effective voluntary measures rather than binding obligations, as seen in other areas of the food industry [64, 65]. Without strong governance mechanisms and independent oversight, public health goals risk being subordinated to commercial interests. Emerging research also highlights evolving OFD platform corporate practices (such as cross-promotion partnerships [66] and co-branded campaigns to share commission on sales and build brand reputation) that expand the reach of less healthy options, further complicating efforts to align platform incentives with public health goals [61].

### Opportunities for Policy Innovation

Although OFD platforms pose clear public health challenges, their scale and integration into everyday routines also present opportunities for public health innovations. Two broad categories of actions could be: mandatory government‑led actions such as labelling and transparency requirements, and platform-led actions such as healthy defaults and restrictions on algorithmic promotions.

#### Mandatory Government‑Led Actions

There is a clear case to extend existing large-chain menu labelling regimes explicitly to OFD platform interfaces, ensuring that when a food outlet is listed on an OFD platform, there is a prerequisite to display calories (or Kilojoules: kJ) adjacent to each item in the same way as in-store menus. Currently, the US considers all digital and non-digital menus in the same way, providing a regulatory blueprint for explicit online coverage [53]. Similar alignment could be advanced in Australia (building on state kJ laws) and reinforced in England through platform-specific guidance or locally relevant government-led programmes or policies. While the emerging evidence from experimental studies [67, 68] simulating the food delivery interface suggests that calorie disclosures result in modest reductions in total energy ordered, this needs to be confirmed in real-world settings for its effectiveness and adoption. While labelling alone will not offset broader platform design features, it represents a promising policy lever.

#### Platform-Led Actions

A growing body of recent evidence demonstrates that platform cues, such as recommendations, default options, rankings, and interface design strongly shape consumer food choices on OFD services [16, 61] and inflating convenience factors [47]. Policy mechanisms could therefore require platforms to display “healthy defaults” and make choice architecture more transparent and accountable. Evidence from simulation studies [67, 68] indicates that labelling, defaults, and availability/placement nudges can result in modest lower calorie or healthier food choices, with combined strategies often outperforming single nudges. A systematic review similarly found digital nudges—especially labelling and availability/accessibility changes—improve dietary choices online [69]. Policymakers could require OFD platforms to offer user-controlled filters (e.g., “lower energy,” “reduced sugar”), default sort orders that surface healthier choices first within the homepage and then within the menu page, and prominence rules limiting the default promotion of less healthy bundles or upsizes. A recent policy review [52] calls for integrating digital food environments into mainstream nutrition policy, moving “offline to online” across labelling, promotion, pricing and retail, precisely where OFD platforms operate.

Considering the substantial and increasing evidence that OFD platforms are reshaping dietary patterns and food purchasing behaviours, we identified a suite of actionable innovations for OFD platforms to support better nutrition outcomes using the NOURISHING framework [70] (see Table 1 below). This framework is a well-established tool designed to operationalise a comprehensive set of policy actions to promote health.

**Table 1:** Policy opportunities for OFD platforms to promote healthier diets using the NOURISHING framework

NOURISHING domain	Potential policy lever	Potential policy action
Nutrition label standards	Require food outlets to share nutrition data and integrate nutrition information into their online menus	Mandatory integration of nutrition information
Offer healthy food	Default sorting by healthier options; reposition reformulated healthy meals to the top of the menu	Healthier menu defaults and algorithmic prioritisation
Use economic tools	Apply price promotion on healthier meal combinations; embed loyalty points for healthy choices	Discounts on healthy food deals
Restrict marketing	Regulate unhealthy food marketing on digital platforms	Extend bans to sponsorships and push notifications
Improve supply	Support food outlets with incentives to offer “healthier food” as defaults	Healthy filter defaults
Set standards in public institutions	Align menus to national dietary guidelines	Partnerships with institutions offering healthy catering
Harness food supply chain	Support food outlets with technical assistance to reformulate food products	Partnerships with food outlets reformulating food products
Inform people	Contextual nudges (example, “Add a veg side”)	Healthy recommendations during and at checkout point
Nutrition advice	AI-driven suggestions aligned to national dietary guidelines	Personalised nudges based on history of food purchases
Governance	Require reporting on consumer exposure to food promotions and sales of discretionary food	A key performance indicator for all OFD services

## Emerging Research and Future Directions

Several technological innovations, such as drone delivery, ghost kitchens, automation and gig-based logistics, are reshaping the OFD service landscape. These innovations may create new public health risks while also presenting novel regulatory challenges. However, empirical evidence on their health, environmental, and equity impacts remains limited.

Drone delivery services are being operated in several countries, including China, the USA, and Australia, and are likely to increase access to unhealthy foods [71, 72]. These technologies are promoted as offering rapid delivery and reduced emissions; however, they also raise concerns relating to privacy, safety, airspace management and susceptibility to extreme weather conditions [73]. Therefore, as the landscape of the OFD sector rapidly changes, more evidence and timely policy measures are needed to monitor and mitigate the potential public health risks resulting from quick access and low cost of unhealthy foods delivered via drone delivery.

Ghost kitchens (or dark kitchens or cloud kitchens) are delivery-only food preparation sites and are expanding rapidly. They offer cost efficiencies for food retailers, but introduce new complexities for regulation, including food packaging waste and food safety. Furthermore, recent studies have shown that consumers have low risk perceptions of such models and are likely to purchase [74, 75]. Further research is needed to understand their contribution to dietary patterns, food safety outcomes, and environmental impact.

OFD platforms rely on gig-based labour models, which provide flexibility but perpetuate precarious employment conditions, income insecurity and unsafe working conditions with limited worker protections [76]. These conditions raise concerns about a range of workforce-related issues. Emerging technologies, including robotics and voice automation, may further reshape labour conditions and consumer experiences [77]. The potential health, well-being and socioeconomic consequences of these shifts remain largely unexamined.

The rapid evolution of online food delivery technologies necessitates a multi-dimensional research agenda [78]. Priority areas include [1] assessing the health and environmental impacts of innovations such as drone logistics, robotics, and ghost kitchens; [2] evaluating how platform design and business models influence dietary behaviours and food access; [3] examining the long-term implications of automation and gig-based employment for worker health and labour equity; and [4] investigating the ethical, privacy and equity considerations of AI-enabled personalisation. These evidence gaps must be addressed to develop policy frameworks that balance technological innovation with sustainability, public health objectives, and equitable access to healthy food.

## Conclusion

OFD platforms have rapidly become embedded in everyday food practices, reshaping how many people access and purchase food. Current evidence indicates that these platforms predominantly promote energy-dense, nutrient-poor foods and embed design features that prioritise convenience over health. While evidence on their potential effects on dietary behaviours are relatively consistent, their impact on physical activity requires stronger empirical evidence. A key caveat of this review is that the evidence base on OFD platforms and their impacts on diet, physical activity and broader health and societal outcomes remains underdeveloped globally and is particularly limited in non-Western contexts. However, where available and published in English, we have added findings from non-Western countries throughout the review. As illustrated in our recent paper, to ensure that OFD platforms support rather than undermine population health, policy frameworks must better reflect the realities of digital food environments [79]. Strong government-led actions are needed to integrate OFD services into national nutrition and physical activity policies, enforce existing nutrition labelling requirements, restrict digital marketing of unhealthy foods, incentivise active delivery modes such as cycling and embed health impact assessments into platform governance. At the same time, research by public health agencies and academic researchers must accelerate to quantify OFD service-attributable risks, evaluate industry accountability and assess compliance with government regulations. Coordinated action and evidence-driven innovation are essential to ensure that digital transformations in the food environment support human health and well-being.

## Key References

- Gupta A, Sacks G, Cameron AJ, Huggins CE, Peeters A, Backholer K, et al. Use of online food delivery services among adults in five countries from the International Food Policy Study 2018–2021. Preventive Medicine Reports. 2024;43:102766. https://doi.org/10.1016/j.pmedr.2024.102766 .
  - ○ Demonstrates rising prevalence and sociodemographic profiles of users of OFD platforms.

- Gupta A, Fraser K, Cameron AJ. Rethinking public health in the digital food era. International Journal of Behavioral Nutrition and Physical Activity. 2026;23(1):52. https://doi.org/10.1186/s12966-026-01920-1.
  - ○ Demonstrates why OFD platforms require mandatory regulation to protect public nutrition and health.

- Gupta A, Backholer K, Huggins CE, Leung GKW, Bennett R, Peeters A. Consumers acceptability of using screen capture methods to capture marketing strategies on online food delivery platforms- A qualitative study. Public Health Nutrition. 2025:28(1):e88. https://doi.org/10.1017/s1368980025000515
  - ○ Demonstrates real-time exposure of consumers to unhealthy marketing tactics on the OFD platforms and the drivers of online food choices.
- Gupta A, Huggins CE, Salashoornezhad S, Bennett R, Backholer K, Peeters A. Digital marketing techniques within online food retail platforms: A scoping review. BMC Medicine. 2026;24(1):30. https://doi.org/10.1186/s12916-025-04553-6
  - ○ Demonstrates a range of persuasive digital marketing tactics employed by OFD platforms to preferentially promote nutrient-poor foods and influence food choices.

## Data Availability

No datasets were generated or analysed during the current study.
